# Clinical validation of free breathing Respiratory Triggered Retrospectively Cardiac Gated Cine Steady-State Free Precession (RT-SSFP) imaging in sedated children

**DOI:** 10.1186/1532-429X-15-S1-O98

**Published:** 2013-01-30

**Authors:** Rajesh Krishnamurthy, Amol Pednekar, Esben Vogelius, Lamya A Atweh, Zili D Chu, Wei Zhang, Prakash Masand, Shiraz A Maskatia, Shaine A Morris, Raja Muthupillai

**Affiliations:** 1 Pediatric Radiology, Texas Children's Hospital, Houston, TX, USA; 2 Departments of Pediatrics (Cardiology), Texas Children's Hospital, Houston, TX, USA; 3 MR Research, Philips Healthcare, Houston, TX, USA; 4 Department of Radiology, St. Luke's Episcopal Hospital and Texas Heart Institute, Houston, TX, USA

## Background

Cine steady-state free precession (SSFP) is the preferred sequence for ventricular function evaluation. However, SSFP demands uninterrupted RF excitation to maintain steady-state (SS) during suspended respiration. This is feasible in adults who can perform breath-holding (BH), but is difficult to accomplish in sedated or uncooperative children. To overcome this, many pediatric groups routinely perform multi-NSA acquisitions (MN) during free breathing. In this work, we validate a respiratory triggered (RT) SSFP sequence that drives the magnetization to steady-state before commencing cardiac gated cine acquisition in sedated pediatric population.

## Methods

This prospective study was performed on 12 sedated children with congenital heart disease (age: 7±3 yrs) with IRB approval.

### MRI Acquisition

All imaging was performed on a commercial MR scanner (1.5 T, Achieva, Philips Healthcare). Identical imaging parameters were used for MN and RT cine SSFP sequences [1] covering both the ventricles in short-axis (SA) orientation (TR/TE/flip angle: 3/1.5/60; acquired voxel size: 1.5-1.9 x 1.5-2.1 x 7-8 mm3; SENSE acceleration factor: 2; temporal resolution: 30-45 ms; acquisition time: 8-10 RR intervals/slice).

### Data Analysis

**Figure 1:**
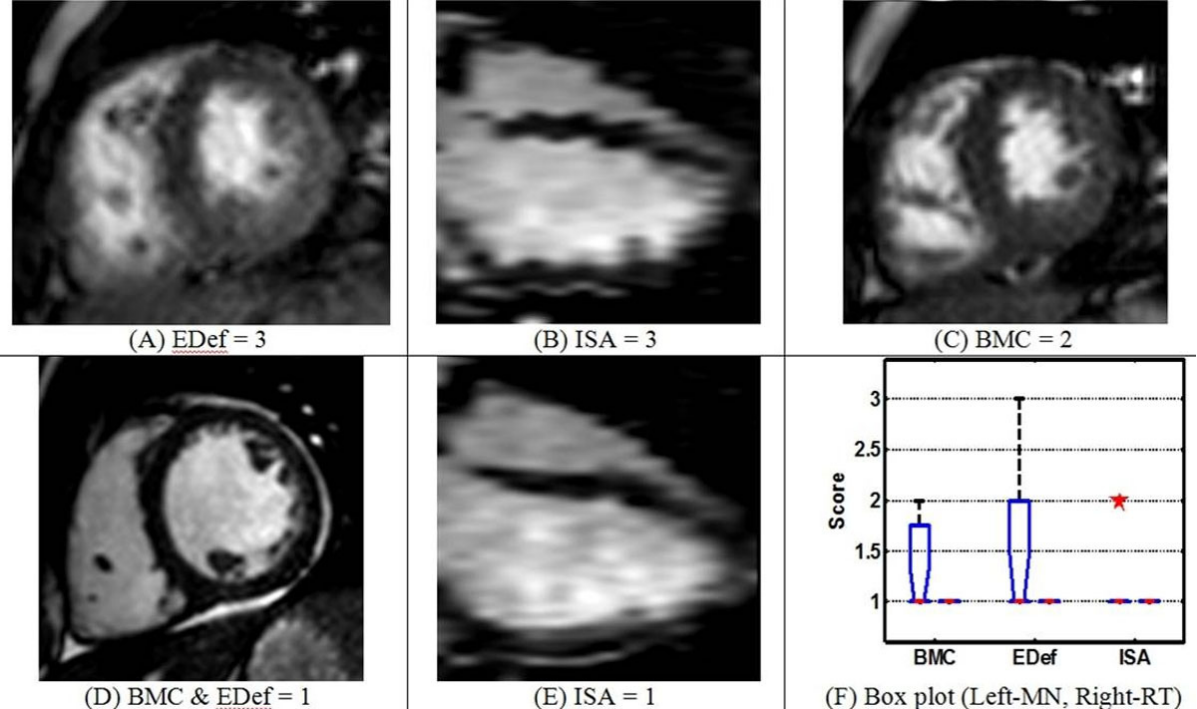
(A-E) Representative images for the clinical scores. The clinical scoring system was as follows: A. Blood-myocardial contrast (BMC): 1-like SSFP, 2-like turbo GRE, 3-worse than turbo GRE; B. Endocardial edge definition (EDef): 1-excellent, 2-good, 3-poor; C. Inter-slice Alignment (ISA): 1-Excellent alignment, 2- <2 slices misaligned, 3- >2 slices misaligned. BMC score reflects quality of the steady state, and EDef score indicates intra-slice motion blurring. ISA was determined by visualizing the SA stack as a volume. (F) Box plot for clinical scores depicts spread of the scores.

Image quality assessment (Figure 1) and quantitative volumetric analysis was performed on the datasets by independent blinded users. One-sided Wilcoxon signed rank test and Box plot analysis were performed to compare the RT-SSFP clinical scores against MN-SSFP. Bland-Altman (BA) analysis was performed on LV and RV volumes.

## Results

The clinical scores for RT-SSFP were consistently better than MN-SSFP (Figure 1). BA analysis (Table. 1) indicates that variability between RT and MN acquisitions is comparable to inter and intra-observer variability reported in the literature [2]. In sedated children, the BMC (p=0.06) and EDef (p=0.06) scores were significantly better for RT-SSFP than MN-SSFP while ISA (p=0.5) was comparable. The ISA due to inconsistent respiration level leads to inconsistent estimation of basal LV volume which can explain the variability in EDV and ESV using two different acquisitions. Total score (with equal weights to each clinical score category) was significantly better for RT compared to MN (p=0.03). Total scan duration for SA stack using RT-SSFP (4.1±0.97 min) was slightly shorter than MN-SSFP (4.6±0.9 min) acquisitions.

**Table 1:** 

Table 1RT vs. MN (n = 12)						
	LV			RV		

	EDV ml	ESV ml	EF %	EDV ml	ESV ml	EF %

Bias	4	2.2	-0.8	3	-0.4	1.1

Limit of Agreement	8.6	6.6	8.2	17.5	10.8	9.1

## Conclusions

The free breathing RT-SSFP sequence allows clinically diagnostic images in sedated children without any penalty for total scan time, and offers improved myocardial blood-pool contrast, and edge definition when compared to MN-SSFP.

## Funding

None.

